# Towards a Microbial Thermoelectric Cell

**DOI:** 10.1371/journal.pone.0056358

**Published:** 2013-02-26

**Authors:** Raúl Rodríguez-Barreiro, Christian Abendroth, Cristina Vilanova, Andrés Moya, Manuel Porcar

**Affiliations:** 1 Cavanilles Institute of Biodiversity and Evolutive Biology, Universitat de València, València, Spain; 2 Unidad Mixta de Investigación en Genómica y Salud, Centro Superior de Investigación en Salud Pública (CSISP), València, Spain; 3 Fundació General de la Universitat de València, València, Spain; University of Nottingham, United Kingdom

## Abstract

Microbial growth is an exothermic process. Biotechnological industries produce large amounts of heat, usually considered an undesirable by-product. In this work, we report the construction and characterization of the first microbial thermoelectric cell (MTC), in which the metabolic heat produced by a thermally insulated microbial culture is partially converted into electricity through a thermoelectric device optimized for low ΔT values. A temperature of 41°C and net electric voltage of around 250–600 mV was achieved with 1.7 L baker’s yeast culture. This is the first time microbial metabolic energy has been converted into electricity with an *ad hoc* thermoelectric device. These results might contribute towards developing a novel strategy to harvest excess heat in the biotechnology industry, in processes such as ethanol fermentation, auto thermal aerobic digestion (ATAD) or bioremediation, which could be coupled with MTCs in a single unit to produce electricity as a valuable by-product of the primary biotechnological product. Additionally, we propose that small portable MTCs could be conceived and inoculated with suitable thermophilic of hyperthermophilic starter cultures and used for powering small electric devices.

## Introduction

Both developed and fast growing developing countries exhibit steadily growing energy demands. Taking into account the limited nature of oil, coal and gas reservoirs, this could obviously lead to a shortage of standard (fossil) fuels in the relatively near future. The lack of sustainability of current fossil-centered energy strategies, as well as the recent extremely serious accident at Fukushima Daiichi power facility [1] have increased the concerns about the economic and environmental consequences of relying on these energy sources, leading to some dramatic shifts in energy policies, like in Germany [2]. It is widely accepted that massive fossil fuel consumption, which results in the production of nine billion metric tons of atmospheric carbon per year [3], is at least partially responsible for current global warming. Therefore, alternative non-fossil non-nuclear technologies are seen as promising, albeit not fully competitive. Among these, biomass-based energy has been suggested as one of the most promising technologies for renewable energy production [4], [5]. Biomass from crops; urban, industrial or agricultural wastes; green algae, cyanobacteria or other microbial cultures, are renewable organic resources that are suitable for energy production in the form of biofuels (mainly, but not limited to, bioethanol and biodiesel), and electricity.

Besides lignocellulosic combustion-based power production, a biological system allowing direct conversion of biomass into electricity already exists: a broad range of organic substances can be oxidized by electrogenic bacteria, which transfer electrons to an anode in a simple device known as a Microbial Fuel Cell (MFC). At the cathode, other useful products can be generated, including hydrogen, methane, and hydrogen peroxide [6], [7], [8]. The electric yield of MFCs has increased dramatically in recent years, mainly by increasing the ratio of the area of the electrodes/volume in the reactor, with best yields reaching up to 2–7 W/m^2^. A moderate MFC unit, of about 1 L, can produce enough electricity to power a small propeller for more than one year [9]. However, MFCs seem to work better at small scales, as scaling-up faces important challenges [9].

Many bacterial species have been reported to display electroactive properties, including members of common genera such as *Clostridium*, *Pseudomonas*, *Geobacter* or *Shewanella*. A few eukaryotic microorganisms have been assayed for power production in MFCs. Baker’s yeast *Saccharomyces cerevisisae* has proven able to transfer electrons to an anode in two independent studies [10], [11] with moderate efficiency. In both reports, researchers found net voltage values of about 0.33 V for 1 L reactors.

To date MFCs are still the only direct method to microbiologically convert biomass into electricity. Nonetheless, there is possibly another non-fuel alternative. Since microbial growth is an exothermic process, it produces heat, which is a by-product that usually goes unnoticed in lab-scale cultures but which has a strong impact on the design and performance of industrial-scale microbial fermentations. Almost 90% of the heat produced in a microbial fermentation is reported to be metabolic heat; and almost all this heat is removed through forced heat exchange [12].

The thermoelectric or Peltier-Seebeck effect is the direct conversion of electric voltage to temperature differences (Peltier effect) and vice-versa (Seebeck effect). Theoretically, an electric current would be produced by coupling an exothermic microbial culture with an endothermic reaction –or, alternatively, a heat sink– through a thermoelectric cell. If the thermal energy from exothermic microbial cultures could be turned into electricity efficiently, power-producing devices could be designed and coupled to existing microbial reactors within a range of applications (alcoholic fermentations, bioremediation, waste treatment, autotrophic thermal aerobic digestion ATAD, etc.).

Here, we report the characterization of the first Microbial Thermoelectric Cell, a bioreactor specifically designed for power production through a completely different mechanism than that operating in MFCs: the thermoelectric effect. Our results might contribute to providing a new scenario for the future development of microbial-based cellular electricity facilities, which might be useful for local electric production and heat recycling in a wide range of biological processes.

## Materials and Methods

### Construction of the MTC

**Figure 1:**
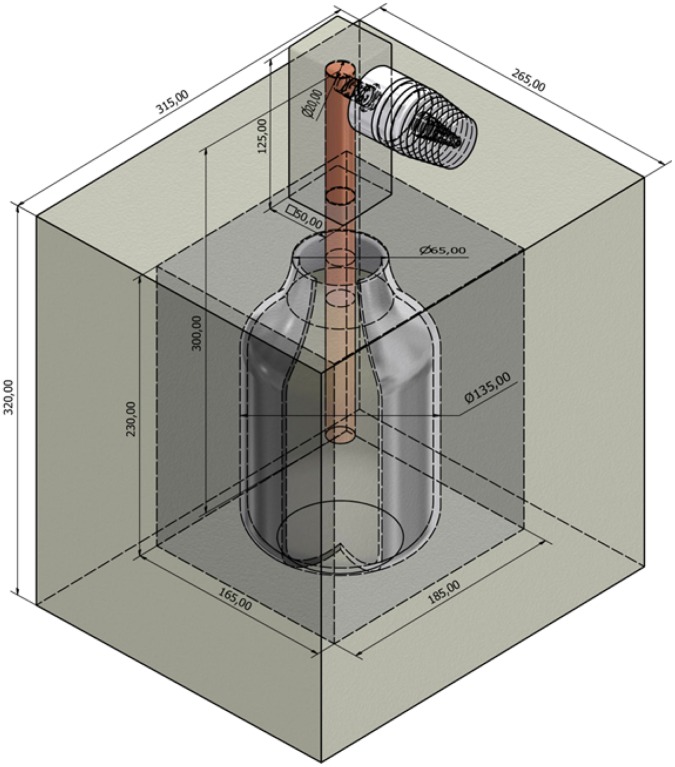
Schematic drawing of the Microbial Thermoelectric Cell (Auto-CAD). All dimensions are given in mm.

In order to implement a thermoelectric-based power generator, a reactor was designed able to i) sustain microbial growth; ii) remain thermally isolated on most of its surface; and iii) efficiently transfer heat through a relatively small area to a thermoelectric device. One of us (M. Porcar) had previously designed an LCC (Liquid Culture Calorimeter) for microbial growth, suitable for fine recordings of internal temperature changes through a thermocouple [13]. Based on the LCC, we conceived a Microbial Thermoelectric Cell (MTC hereon) to produce power from a microbial culture by the Peltier-Seebeck effect. Figure 1 shows the structure of the MTC. The core of the reactor is a 1.9 L glass container from a commercial vacuum flask. The flask was placed inside an expanded polystyrene (EPS) box and the gap filled with a polyurethane foam spray (Silicex Fischer, Fisher Ibérica, Tarragona, Spain). The box was then inserted into a second EPS isolation box. The upper part of the MTC was drilled and a cupper bar (20 mm in diameter) inserted through the hole. The upper part of the cupper bar was adapted in order to allow a TE-Power Probe thermal harvester (MicroPelt, Germany) to be screwed through a 1/4″ Whitworth thread (DIN 2999, JIS B0203, ISO 7/1). TE-Power Probe is a prototype of an integrated proximity thermoharvester designed to replace primary batteries in wireless systems operating in duty cycle mode. The key element of the TE-Power Probe is the MPG-D751 thermogenerator, which produces electricity from a rather low gradient of temperature. The TE-Power Probe is originally designed to attach to a heat source in the shape of piping that carries a hot fluid, and heat is dissipated through an aluminum heat sink, with the resulting temperature gradient allowing power production by the MPG-D751 thermogenerator. In our experiments, temperature changes in different parts of the Probe were measured by PT-100 sensors. Since the TE-Power Probe is specifically designed to operate using natural convection to ambient air, we mounted it horizontally, as suggested by the manufacturer.

The two EPS isolation layers of the MTC were shaped so the round bottom of the vacuum flask would fit. The flask bottom was placed conveniently close (20 mm) to the bottom of the MTC in order to allow stirring by a magnetic stirrer. When recordings were to be taken, the MTC was first filled with 1.8 L of medium; a small magnet was added; the MTC was placed inside a standard laboratory magnetic stirrer set at low speed (600 rpm); the inoculum was then added, and the cupper bar with the screwed TE-Power Probe finally set in place. This configuration was modified for characterization purposes in some experiments, as described in sections 2.4 and 2.5.

### Yeast Strains, Media and Culture Conditions

The following six diploid *S. cerevisiae* strains, from the wine industry or genetic modifications thereof, were used: EC118, L2056, 3aS2Δ, T73, D170, and TTRX2. All the strains were kindly provided by Prof. Emilia Matallana (IATA, Valencia, Spain). In order to assess their exothermic abilities, independent cultures were set in filter-sterilized YPD (20 g/L peptone, 10 g/L yeast extract, with 18% sugar), and the internal temperature of the cultures (grown overnight in non-isolated Erlenmeyer flasks) was continuously measured. Thermotolerance was assessed by growing the strains at 30, 37 and 41°C. After an overnight incubation under low stirring, the OD_600_ of the six overnight cultures was measured.

For standard experiments after strain selection, the filter-sterilized 18% sucrose YPD was inoculated with 1∶50 of an overnight yeast pre-culture grown at 30°C, and subjected to low stirring for 120 h.

### Data Acquisition, Monitoring and Recording

The MTC was connected to a PC in order to record internal and external temperatures and the output current provided by the heat harvester TE-Power Probe (Fig. S1).

The internal temperature of the MTC was measured by a thin T-type thermocouple inserted into the microbial culture and connected to a PC through a data logger, as previously described [13]. Another thermocouple recording room temperature was also set in place. The thermocouples were connected to an acquisition card inserted on the data logger, which was connected via a GPIB cable to a PC with an acquisition software that one of us (R. Rodríguez-Barreiro) conceived specifically for this work (Fig. S1). The TE-Power Probe output was also connected to the PC, which yielded two additional temperature recordings by using two Pt-100 sensors (that of the cold and hot sides of the thermogenerator device) and the output voltage. The connections between the thermocouples and the data logger were performed on an ice-water mixture to take into account the unwanted background electric voltage, due to the junction of dissimilar metals in the thermocouple-data logger connection. Finally, a thermocouple was inserted inside the box containing the ice-water mixture in order to verify that the temperature of the datalogger-thermocouple connections was kept at 0°C.

Temperature records (and, when TE-Power Probe was connected, electric power) were taken every 6 minutes throughout the experiment.

### Identification Assay to Estimate Broth Heat Capacity and Global Thermal Resistance of MTC

In order to estimate the heat capacity of the broth (*m·C_p_*) and the global thermal resistance (*R_g_*), the MTC (without TE-Power Probe) was set up under the following conditions: first, an electrical resistance was placed inside the MTC to generate a controlled heat flow, as consequence of the Joule effect induced by an external voltage input through the resistance. Second, the MTC was loaded with room-temperature sterile broth with 1 g/L nipagine supplementation to avoid contamination by yeasts. Broth was subjected to continuous stirring and room temperature was kept constant. Throughout the experiment, broth and room temperatures and the power generated in the resistance were continuously measured. To ensure the initial steady-state conditions (broth temperature equal to room temperature), the system was kept in the off mode for approximately 20 h before applying the input voltage.

### Theory

The equation for the heat flow balance corresponding to the MTC we describe in this work can be stated as follows:


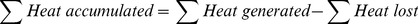
(1)

Heat accumulated is a consequence of the variation in broth temperature. Since there is no forced cooling of the system, heat flow losses are due only to heat transfer from the culture to the environment through both the MTC surface and the TE-Power Probe thermogenerator. For calibration purposes, we first set the MTC to generate a heat flow from an electric resistance placed inside the vacuum flask through the Joule effect. In standard experiments, heat flow was obtained from the metabolic heat as a consequence of microbial growth.

Therefore, the heat flow balance equation can be written for the MTC as follows (a definition of all the symbols used throughout the MTC modelling is available in Table 1):

**Table 1:** Nomenclature used in MTC modelling.

Symbol	Description (units)
*α*	Seebeck coefficient (V/K)
*I*	Electrical current (A)
*m· C_p_*	Whole system heat capacity (J/K)
*m_b_·C_pb_*	Broth heat capacity (J/K)
*m_v_· C_pv_*	Vacuum flask heat capacity (J/K)
*m_w_· C_pw_*	Insulation walls heat capacity (J/K)
*P_J_*	Electrical input power due to the Joule effect (W)
*P_e_*	Electrical power generated (W)
*Q_acc_*	Accumulated heat flow (W)
*Q_C_*	Net heat flow released through the cold side of the thermogenerator (W)
*Q_env_*	Heat flow released to the environment (W)
*Q_H_*	Net heat flow absorbed through the hot side of the thermogenerator (W)
*Q_j_*	Heat flow due to the Joule effect inside of the thermogenerator (W)
*Q_p_*	Heat produced by microbial metabolism (W)
*Q_sC_*	Heat flow produced in the cold side of the thermogenerator due to the Seebeck effect (W)
*Q_sH_*	Heat flow produced in the hot side of the thermogenerator due to the Seebeck effect (W)
*Q_t_*	Heat flow loss due to the natural thermal conduction established between both sides of the thermogenerator (W)
*Q_Th_*	Heat flow absorbed from the broth through the cupper bar (W)
*R*	Electrical resistance (Ω)
*R_Cu_*	Thermal resistance of the cupper bar (K/W)
*R_g_*	Global thermal resistance of the MTC (K/W)
*R_i_*	Internal electrical resistance of the thermogenerator (Ω)
*R_Load_*	Electrical resistance connected between the terminals of the thermogenerator (Ω)
*R_Sk_*	Thermal resistance of the heat sink (K/W)
*R_th_*	Thermal resistance of the thermogenerator (K/W)
*T_b_*	Broth temperature (K)
*T_C_*	Temperature of the cold side of the thermogenerator (K)
*T_env_*	Room temperature (K)
*T_H_*	Temperature of the hot side of the thermogenerator (K)
*ΔT_th_*	Difference of temperature between the hot and the cold sides of the thermogenerator (K)
*T_v_*	Vacuum flask temperature (K)
*T_w_*	Insulation walls temperature (K)
*V_ext_*	Input voltage (V)
*V_o_*	Voltage output in the terminals of the thermogenerator (V)



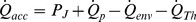
(2)Where *Q_acc_* is the heat flow accumulated in the broth; *P_J_* is the heat flow due to the Joule effect; *Q_p_* is the heat flow due to microbial metabolism; *Q_env_* is the heat flow loss through the MTC surface to the environment; and *Q_Th_* is the heat flow loss through the cupper bar connected to the TE-Power Probe.

### Accumulation Terms

Heat accumulation (*Q_acc_*) in a particular body is determined by the variation in its temperature (*dT_i_/dt*) and by its heat capacity (*m_i_·C_pi_*). In the MTC, heat can be accumulated in the broth (subscript “*b*”), the vacuum flask (“*v*”) and the insulation walls (“*w*”), as follows:
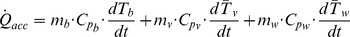
(3)


The MTC is a very simple system with a single sensor to measure the temperature of the broth. Therefore, the equation can be simplified:
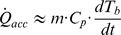
(4)


Where *m·C_p_* represents the whole system heat capacity, deduced from the variation in culture temperature. This parameter can easily be determined under a simplified experimental configuration (described in 2.4) using the model equations described below (section 3.2).

### Loss Terms (I): Heat Flow Loss to the Environment

Energy losses through the MTC walls can be due to the natural heat flow (*Q_env_*) from the warm internal broth to the relatively cool environment. Since insulation materials in the MTC display low emissivity values, radiation losses can be neglected and *Q_env_* can be expressed as follows:
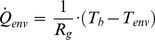
(5)


Where *R_g_* represents the global thermal resistance of the MTC and *T_b_* and *T_env_* are the temperatures of the broth and the environment, respectively. This thermal resistance can be experimentally determined under the same conditions described for *m·C_p_* (see 2.4 and 3.2).

### Loss Terms (II): Heat Flow Loss Through the TE-Power Probe

The global heat flow through the cupper bar (*Q_Th_*) is the same than the heat flow absorbed by the hot side of the thermogenerator cell (*Q_H_*) and is composed of: (i) a spontaneous flow due to the difference in temperature between the hot and cold sides of the thermogenerator cell, expressed as *(T_H_-T_C_)/R_th_*; (ii) an induced heat flow as a consequence of the conversion of heat to electric power through the Seebeck effect. Then, the heat flow loss through the thermogenerator can be stated as follows [14]:
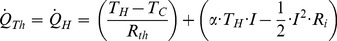
(6)


Where *α·T_H_·I* corresponds to heat absorbed by the thermogenerator due to the Seebeck effect, while the term *1/2·I^2^·R_i_* corresponds to the heat produced as a consequence of the Joule effect, associated to the circulation of the electric current produced through the internal resistance of the thermogenerator. *T_H_* and *T_C_* represent the temperature of the hot and cold sides of the cell, whereas *R_th_* and *R_i_* correspond to its internal thermal and electrical resistance, respectively. *α* is the Seebeck coefficient of the thermogenerator and *I* is the electrical current obtained from the TE-Power Probe.

### Generation Terms (I): Heat Flow Due to the Joule Effect

When an electrical resistance was placed inside the vacuum flask, a heat flow (*P_J_*) was obtained as a consequence of applying an external voltage according to the Joule effect:
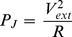
(7)


Where *V_ex_*_t_ is the input voltage and *R* is the electrical resistance.

### Generation Terms (II): Heat Flow Due to Yeast Growth

When the electrical resistance was replaced by a yeast culture, the heat flow was generated as a consequence of the exothermic properties of yeast metabolism. This heat flow, represented as *Q_p_*, was estimated for the different experimental configurations as described below (section 3.3).

Taking all the equations described above together, the general energy balance (Eq. 2) can be written as:
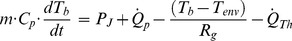
(8)


### Model Equations for the Estimation of m·C_p_ and R_g_

In order to calculate the global heat capacity and the global thermal resistance of the MTC (*m·C_p_* and *R_g_*, respectively), a simplified experimental set up was used, as explained in section 2.4. Briefly, heat flow was induced in the sterile broth by applying a constant input power through a resistance according to the Joule effect. In this experiment, room temperature was kept constant and the TE-Power Probe was not mounted on the MTC. Therefore, *Q_Th_* and *Q_p_* terms (corresponding to the TE-Power Probe and the yeast, respectively) from Eq. 8 are null, so it can be written as the following first-order EDO:
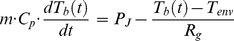
(9)


A first-order EDO is mathematically characterized by its gain and its time constant, which can be estimated manually or with a standard mathematical software from experimental data. In Eq. 9, the gain (*R_g_*) and the time constant *(m·C_p_·R_g_)* were estimated from the experimental values of *T_b_* and *P_J_*.

### Estimation of Heat Yield Due to Yeast Metabolism and Calculation of the Electrical Power Generated

Heat yield due to yeast metabolism (*Q_p_*) was estimated from Eq. 8, where the term *P_J_* is null since no electrical resistance was set up inside the flask:
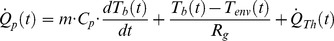
(10)


In the assays where the TE-Power Probe was not included, the term *Q_Th_* (the broth heat lost through the cupper bar) is null, so *Q_p_* was calculated from the experimental data of *T_b_* and *T_env_* using the estimations of *m·C_p_* and *R_g_* previously obtained.

When the TE-Power Probe was included, the metabolic heat yield was calculated from Eq. 10, along with the model equations for TE-Power Probe in order to estimate *Q_Th_* (a detailed description of these equations and a schematic representation of associated heat flows is available in Appendix S2 and Fig. S2, respectively). These model equations are dependent on the electrical configuration used in the thermogenerator during the assays. When no load resistance was connected to the terminals of the thermogenerator (no electrical power was taken out), the following equation for TE-Probe was used (for a detailed version of this open-circuit model, see Appendix S1):
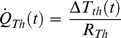
(11)


*ΔT_th_* represents the difference of temperature between the hot and the cold side of the thermogenerator, whereas *R_th_* is the thermal resistance of the thermogenerator.

Voltage output (*V_o_*) of the terminals of the TE-Power Probe, which under this configuration is equal to the voltage generated in the Peltier cell, can be expressed as:
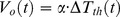
(12)


Being *α* the Seebeck coefficient.

Otherwise, when a load resistance was fitted to achieve the maximum power from the thermogenerator, Eq.11 was replaced by Eq.13 (deduced in the maximum-power model of Appendix S2):


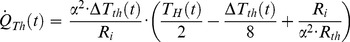
(13)

Where *R_i_* and *R_th_* are the internal electrical and thermal resistance, respectively.

Under this configuration, voltage output (*V_o_*) of the terminals of TE-Power Probe can be expressed as:
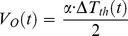
(14) and the maximal power generated can be calculated as follows:
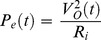
(15)


## Results

### Estimation of Broth Heat Capacity and Global Thermal Resistance of MTC

**Figure 2:**
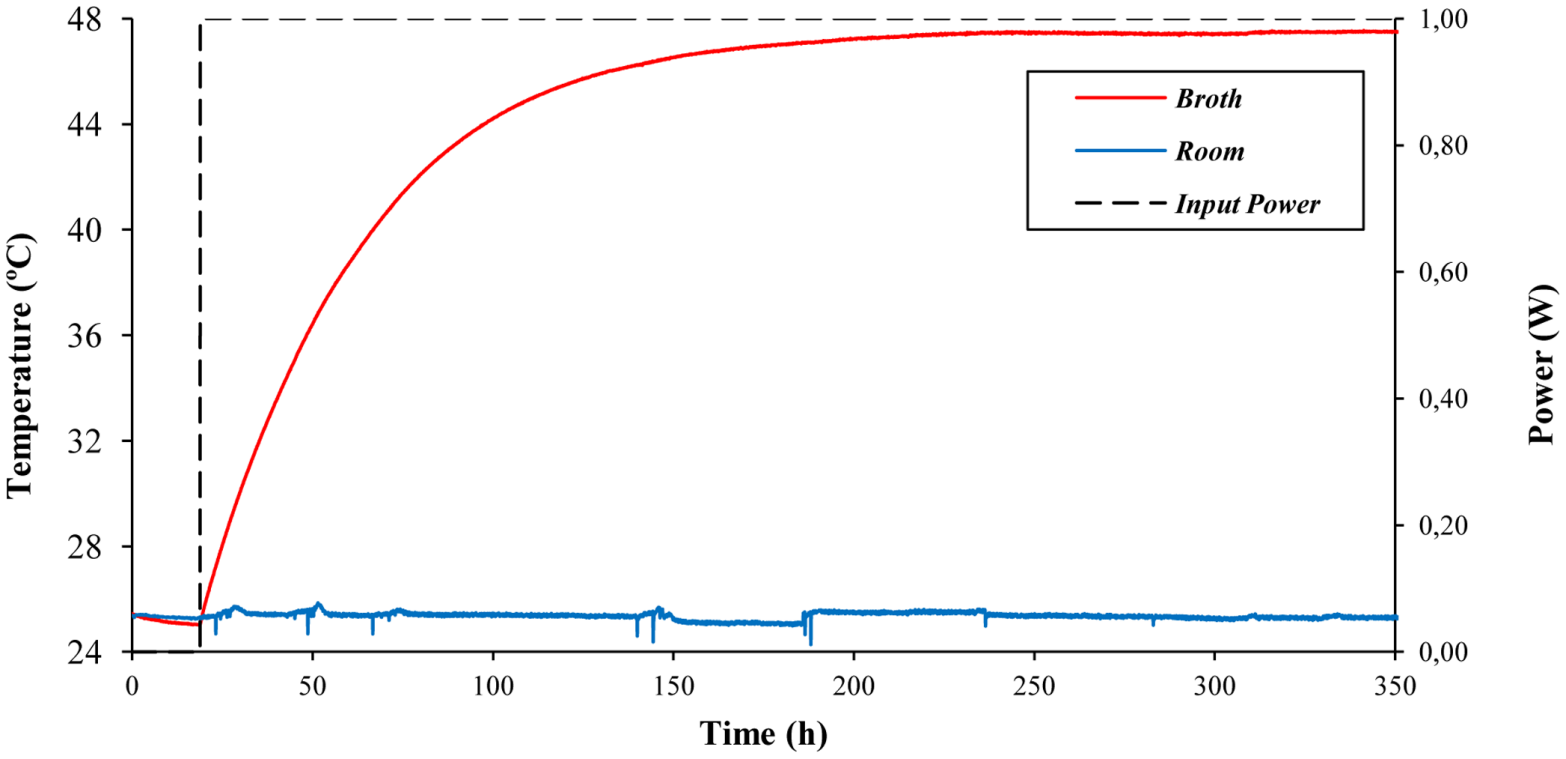
Time course of broth and room temperatures during the identification assay of broth heat capacity and global thermal resistance of the MTC. The experiment was carried out under the conditions described in section 2.4. Recordings of room temperature (blue), broth temperature (red) and input power (dashed line) were taken every 6 min.

In order to characterize the thermal evolution of the MTC prior to the experiments with yeast cultures, an identification assay for *m·C_p_* and *R_g_* was set up as described in 2.4. The time course of broth and room temperature during the experiment is shown in Figure 2. From a steady-state, in which room and broth temperature were the same (25.5°C), a constant input power of 1 W was supplied, and the broth reached a final temperature of 47.5°C. The system gain (meaning the temperature increase divided by the input power) was 22 K/W, and the time constant (the time by which 63% of the temperature increase is reached) was 43.5 h. According to the model equations (see 3.2), the gain represents the global thermal resistance of the MTC, and the broth heat capacity can be obtained by dividing the time constant by the gain. Thus, our estimated values for *R_g_* and *m·C_p_* are 22 K/W and 7118 J/K, respectively.

When the mathematical software was used to estimate *R_g_* and *m·C_p_* from the same experimental data, similar values were obtained (*R_g = _*22 K/W and *m·C_p = _*7100 J/K) with a confidence level of 98.7%.

### Strain Selection and MTC Performance

**Figure 3:**
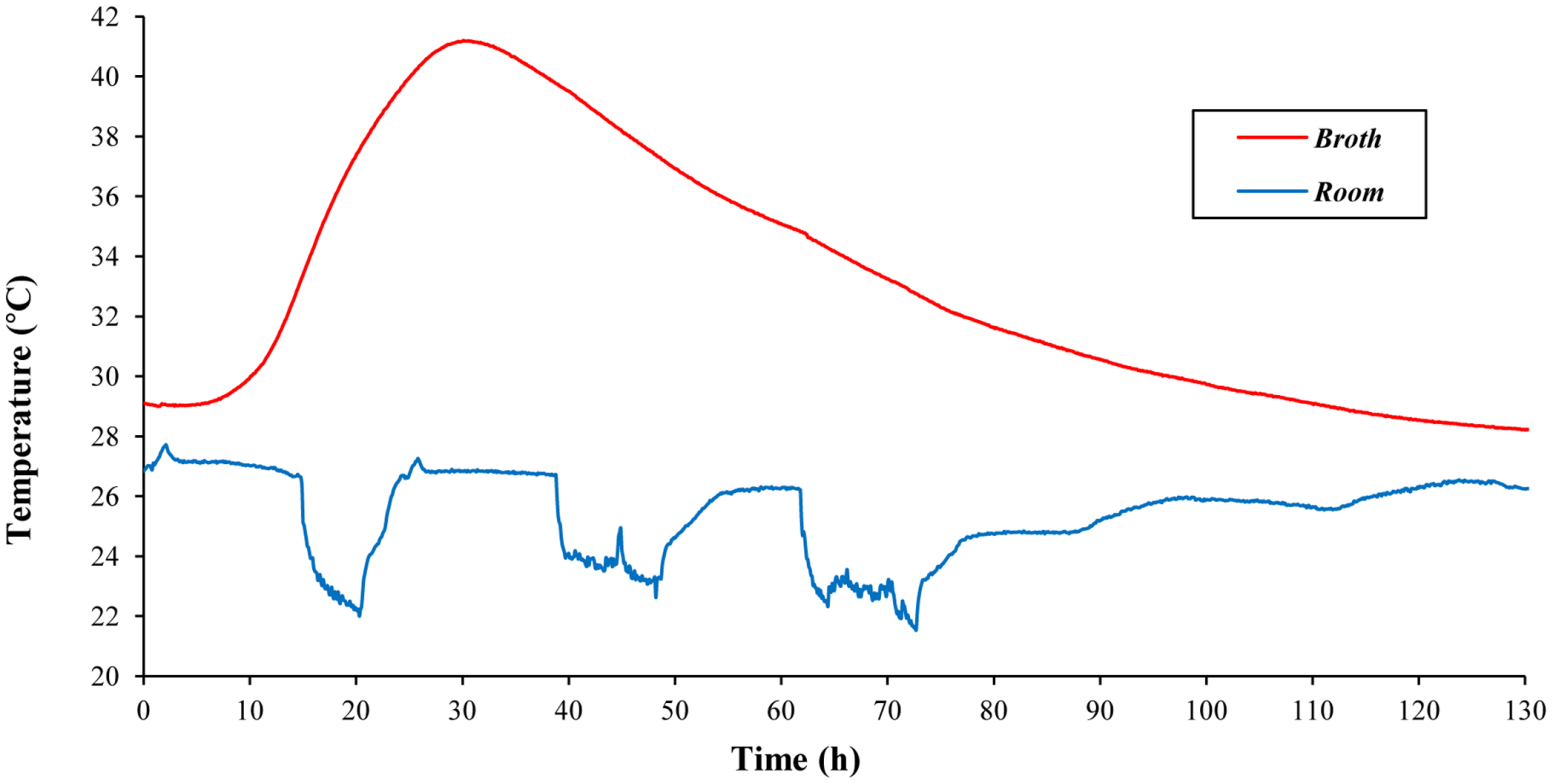
Typical performance of the MTC without TE-Power Probe. Experimental values of broth and room temperature (red and blue lines, respectively) are shown.

All yeast strains exhibited similar performance in terms of exothermic potential and resistance to high temperatures, with strain D170 displaying slightly higher thermoresistance (data not shown). This strain was thus selected for further studies. When yeast strain D170 was inoculated into a pre-warmed 18% sucrose YPD medium and grown in the MTC without the cupper bar and the TE-Power Probe set in place, the internal temperature dropped slowly (about 1°C), stabilized and finally started to rise after 6–7 h. The temperature peaked after approximately 24 h and reached up to 41°C. Figure 3 shows a typical experiment in which the maximum temperature is around twelve degrees higher than the initial temperature of the culture. After the peak, the yeast culture temperatures started dropping and reached the initial temperature after about 70–90 h. Despite the abrupt changes (22°C–27°C) in room temperature as a consequence of switching the air conditioning on and off, the change in the internal temperature of the yeast culture was only mildly affected.

### Estimation of Heat Yield Due to Yeast Growth

**Figure 4:**
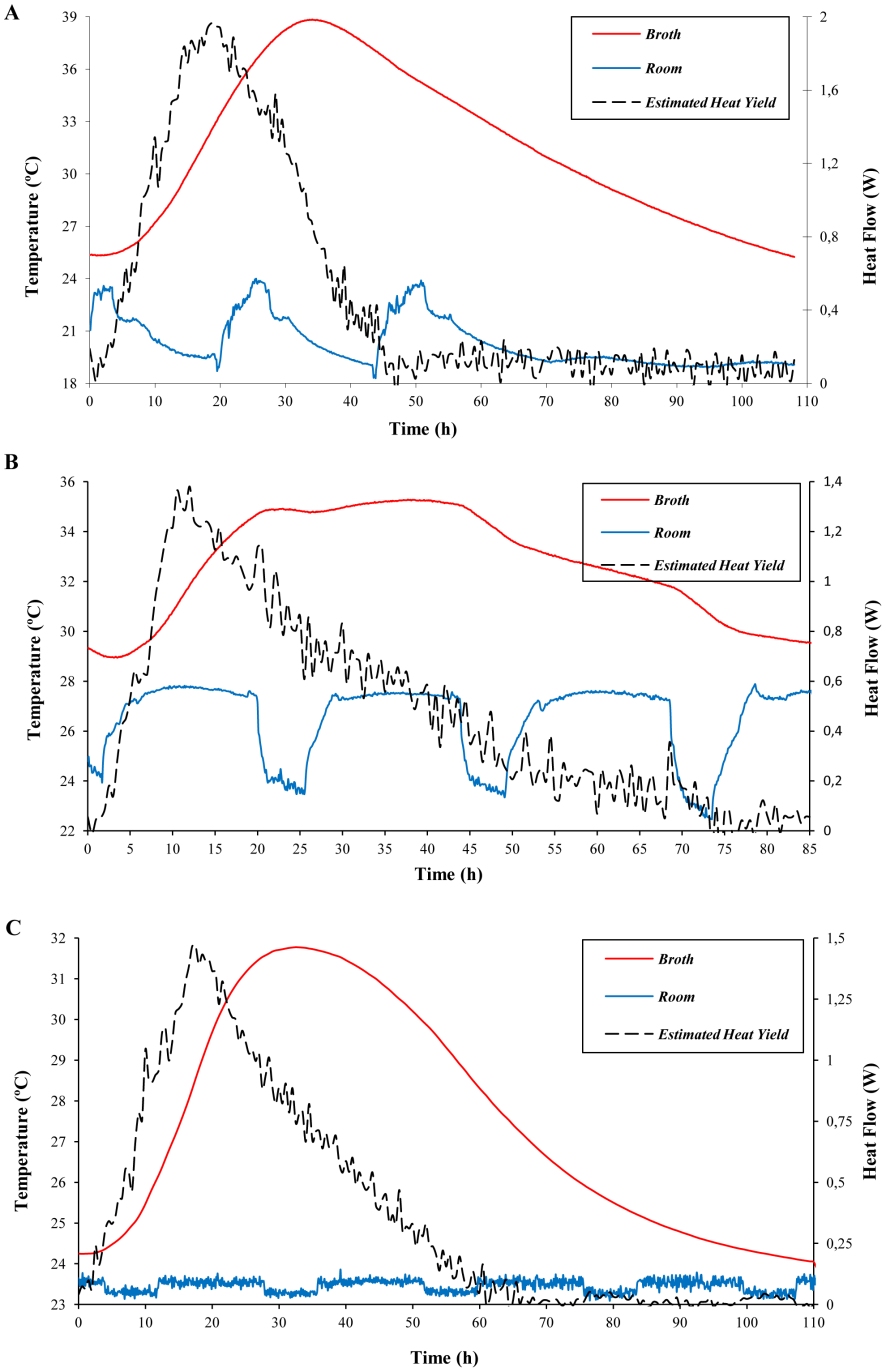
Time course of broth and room temperatures and heat yield due to yeast growth for different MTC configurations: without TE-Power Probe (A), open-circuit (B) and load-resistance (C). Experimental values of broth and room temperature (red and blue lines, respectively) were recorded every 6 min. Heat yield (dashed line) was estimated for each configuration as described in section 3.3.

The heat yield due to yeast growth (*Q_p_*) was estimated as described in section 3.3 for each experimental set up (Fig. 4). In all the experiments, the estimated evolution of *Q_p_* peaked before broth temperature reached its maximum due to the high inertia of the broth (*m·C_p_*). In the assay carried out without TE-Power Probe (Fig. 4A), *Q_p_* reached its maximum (1.96 W) after 20 h and remained above 0.2 W for 40 h. In an open-circuit configuration, maximum *Q_p_* (almost 1.4 W) peaked after 10 h, reaching values above 0.2 W over 50 h (Fig. 4B). Maximum *Q_p_* was obtained earlier in this case because a more concentrated inoculum was used, indicating that, as expected, there is a dependence between initial yeast concentration and time until *Q_p_* maximum. Finally, under a load-resistance configuration, *Q_p_* peaked (with a value of almost 1.5 W) after 20 h (as in the experiment without TE-Power Probe, in which the same initial yeast concentration was used), producing more than 0.2 W for 50 h (Fig. 4C). Our data show that when the TE-Power Probe was inserted, lower *Q_p_* values were estimated from experimental data. In accordance, total energy generated by yeast metabolism, calculated as the area below the curve of *Q_p_*, was higher in the experiment carried out without the TE-Power Probe (194,7 kJ) in comparison with those configurations in which it was included (144,4 and 145,4 kJ for the open-circuit and the load-resistance set up, respectively). This might be due to the effect of the cupper bar on effective broth stirring, which might be lower and therefore affect yeast growth.

### Electricity Production with the MTC

**Figure 5:**
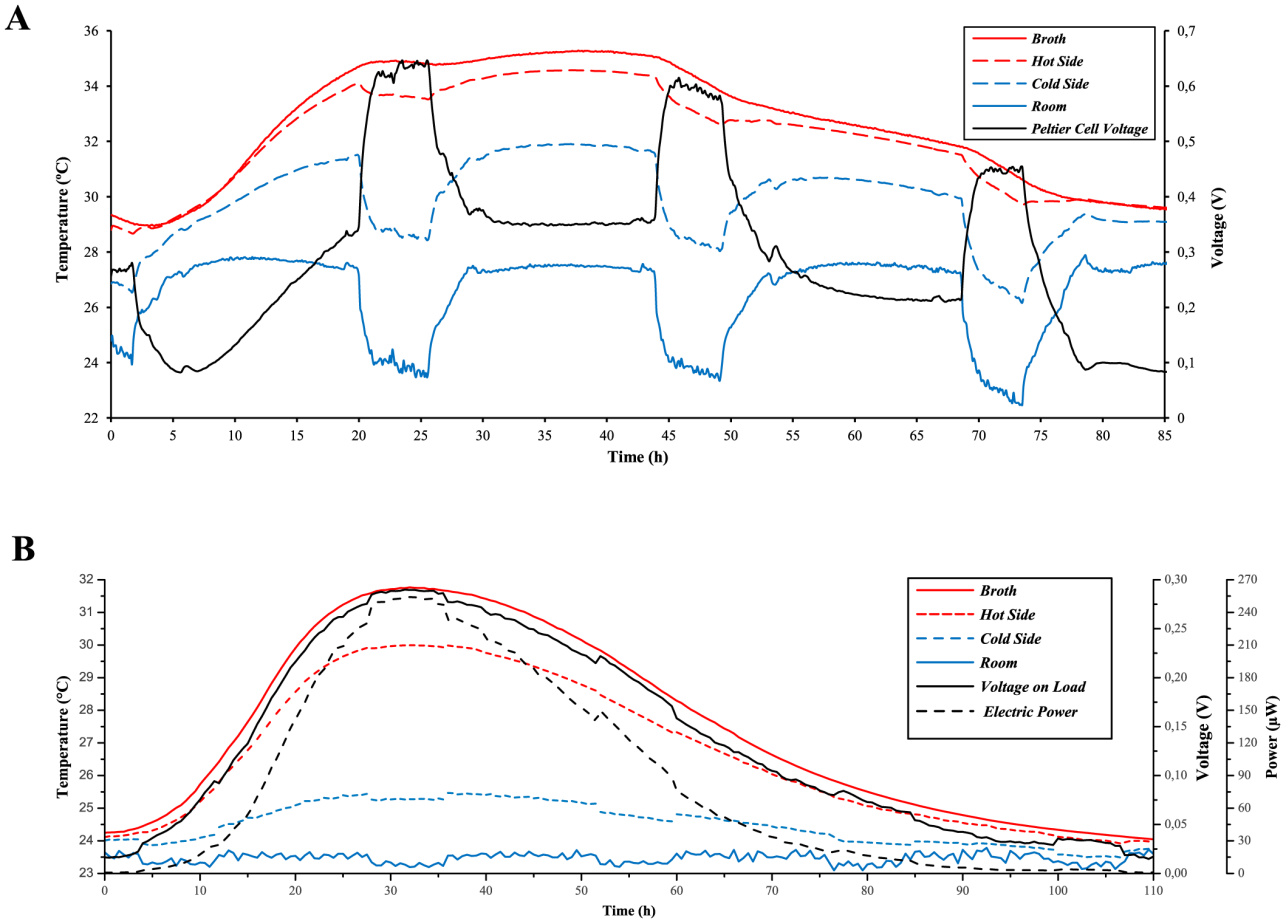
Electricity production by MTC under open-circuit (A) and load-resistance (B) configurations. The experimental temperature values of broth (red), room (blue), and thermogenerator hot and cold sides (red and blue dashed lines, respectively) are shown along with the evolution of voltage and power output (black continuous and dashed lines, respectively).

Under the MTC insulation conditions assayed, the metabolic heat produced by strain D170 was partially transformed into electricity through the TE-Power Probe thermal harvester. When the TE-Power Probe was mounted in the yeast-culturing MTC under open circuit conditions, the internal temperature of the culture increased up to about 35°C and remained higher than 32°C for about 54 h (Fig. 5A). Under these conditions, electric voltage yielded around 250 mV (net value) for a two-day period, with significant, lower room temperature-associated peaks of about 350–600 net mV (Fig. 5A). The same experiment was performed under load resistance conditions (330 Ω, the same as that for the MPG-D751 thermogenerator) and produced an internal temperature peak of about 32°C, with the culture being hotter than room temperature (which was constant in this experiment) for a period of 110 h (Fig. 5B). Under these conditions, a maximum of 290 mV were obtained on the electrical load resistance, corresponding to around 580 mV generated in the Peltier cell (Eq. 15). The maximum power obtained, corresponding to the maximum *ΔT* values, reached around 255 µW (net value).

The energy conversion yield was calculated for this latter experiment as the total electrical power generated (33.1 J) divided by the total heat energy produced by the yeasts (147.44 kJ, as calculated from the estimated heat yield represented in Fig. 4C). The resulting value, 0.022%, was low, as expected from the poor efficiency of heat-harvesting devices such as the TE-Power Probe. Notwithstanding, it allowed the production of significant amounts of electrical power from relatively moderate values of *ΔT.*

## Discussion

The results presented here clearly indicate that the exothermic nature of microbial growth can be exploited when transformed into significant electric voltage. We have designed and constructed the first Microbial Thermoelectric Cell, which consists of a simple, thermally insulated reactor, with a small heat exchange surface on which a thermoelectric prototype thermal harvester, equipped with a MPG-D751 thermogenerator device (TE-Power Probe) is mounted. The chosen thermogenerator is optimum for relatively high efficiencies in electric production at low *ΔT* values, such as those existing between an insulated yeast culture (41°C, under our conditions) and room temperatures. With a medium size MTC (smaller than two liters), we typically obtained 150–600 mV. These values are similar to those obtained with yeast-based MFCs for which net voltage values of about 0.33 V for 1 L reactors have been reported [10], [11]. It is noteworthy that MFCs and MTCs work on a totally different basis –albeit theoretically compatible– as MFCs produce electricity from direct microbial-mediated electron transfer from organic matter oxidation to an anode; whereas the MTC partially transforms metabolic thermal energy into electricity by the Seebeck effect. As it is also the case for MFCs, MTCs could be combined with other microbial processes. Baker’s yeast *S. cerevisiae* was used for our MTC due to its well-known exothermic growth under a range of different conditions. Indeed, any other microbial culture resulting in important heat production, such as ethanolic fermentation (beer, bread, wine, biofuels), auto thermal aerobic digestion (ATAD) or hydrocarbon-polluted soil bioremediation and bioaugmentation, could be coupled with MTCs into a single unit, with electricity production as a valuable sub-product of the main biotechnological purpose. In fact, metabolic heat is often seen in industry as an undesirable sub-product of large-scale microbial fermentations, and cooling facilities are often needed in order to maintain an optimum broth temperature [12], [15]. The conversion, albeit partial, of this heat into electricity would both help to control internal temperatures in biotechnological processes and contribute to energy savings by cogeneration. Interestingly, our results suggest that heat production through metabolic growth and heat flow through a thermogenerator can be tuned in such a way that no energy is needed to heat the broth up for microbial growth nor to cool it down in order to avoid excessive temperatures, known to abruptly stop the fermentation process.

It seems reasonable to predict that, in addition to yeast, other cultures might be suitable as heat producers in an MTC. For example, naturally-occurring thermophilic and hyperthermophilic bacteria, such as *Bacillus coagulans*, *Bacillus licheniformis* or many *Geobacillus spp.* strains, many of which can be isolated from extreme environments such as deep oil wells and the optimal growth of which is 50–60°C. Additionally, these bacteria are reported as able to heat their own culture up to 50–55°C [16]. The perfect candidate for MTC should meet the following criteria: (i) thermoresistant; (ii) strong exothermic ability; (iii) rapid and easy growth; and (iv) an ability to grow and degrade high concentrations of carbon sources.

In the MTC we designed, the “cold side” of the system was an aluminum heatsink. In order to optimize electricity yield by increasing *ΔT*, a biological cooling system could theoretically be implemented, rather than simple convection-driven heat loss. In fact, methanogenic archaea have been reported to exhibit endothermic growth [7]. Although it is uncertain whether endothermia is a result of particular growth or of heat loss due to gas evaporation from the culture, the fact is that these microorganisms could be combined with those producing heat through a thermoelectric element in order to increase electricity production. These archaea have optimal growth at temperatures of around 37°C, and this implies that the whole system should be finely tuned in order to regulate heat transfer across the thermoelectric element, allowing optimal microbial growth while maintaining as high a *ΔT* as possible.

The surface:volume ratio of microbial fermentors is a critical factor affecting heat loss to the environment and thus internal temperature of the culture. Although standard lab-scale microbial cultures produce heat, most of it is lost to environment due to high surface to volume ratio, resulting in the absence of any noticeable increase in internal temperature. However, large, production-scale bioreactors have been characterized thermodynamically and proved to work nearly adiabatically due to much lower surface to volume ratio compared to laboratory-scale non-insulated bioreactors [12]. The results presented here, together with previous reports on medium-scale liquid culture calorimeters [13], demonstrate that relatively small liquid cultures can also work almost adiabatically, provided proper insulation is provided and significant autothermal growth can be achieved. This implies that small portable MTCs for electricity production could be envisaged, since most of the metabolic heat from microbial growth can be stored inside the MTC. These small thermoelectric cells could theoretically be used to power small electric devices. However, in order for MTCs to display higher electric yields, optimization of the thermoelectric elements should take place. Indeed, only 0.5–8% of the total heat flow is usually transformed into electricity through the thermoelectric plates. Interestingly, only 12% of the maximum theoretical efficiency is achieved in the best thermoelectric devices today [17], so there is still room for significant improvement in the optimization of this technology. There has been a dramatic increase in research into high efficiency thermoelectric devices in recent decades, with reports of significant improvements in ZT values, design optimization, and development of alternative materials. As proposed by [17], “TE solid-state heat engines could well play a crucial role in addressing some of the sustainability issues we face today”.

Other heat harvesting methods, such as absorption heat transformers or organic Rankine cycle, have been reported previously [18], [19]. However, these systems are space-consuming and involve mobile parts that require continuous maintenance. In contrast with these, solid-state thermoelectric systems are small, require almost no maintenance, and display high adaptability to a range of industrial designs [17].

In conclusion, this is the first report of microbial metabolic energy being converted into electricity with an *ad hoc* thermoelectric device, i.e., the Microbial Thermoelectric Cell. Our results show that even small volumes of broth are able to exhibit significant autothermal performance and produce electricity when properly insulated and set in such a way that heat exchange is minimized over the whole surface, except the small area on which a (prototype) thermal harvester is mounted. Although the electric power we obtained was rather low, this work may contribute towards a novel strategy to harvest excess heat produced by the biotechnology industry, particularly if ongoing research into thermoelectric materials and design finally yields high efficiency thermoelectric devices.

## Supporting Information

Figure S1 **Schematic drawing of MTC data-recording system.** Dashed lines represent thermocouple connections measuring the temperature of the broth (*T_b_*), the temperature of the hot and cold sides of the thermogenerator (*T_H_* and *T_C_*, respectively), and the room temperature (*T_env_*); whereas continuous lines represent voltage measurements corresponding to the thermogenerator (*V_th_*) and the electrical resistance (*V_r_*).
(TIF)

Figure S2 **Schematic drawing of heat flows and resistances within the thermogenerator cell.** Symbols used are in accordance with the nomenclature summarized in Table 1.
(TIF)

Appendix S1 **Thermogenerator cell (MPG-D751) general equations.**
(DOCX)

Appendix S2 **TE-Power Probe model description.**
(DOCX)
