# Supplementary figures and images for: Towards a Microbial Thermoelectric Cell
Source: PLoS One. 2013 Feb 26;8(2):e56358. doi: 10.1371/journal.pone.0056358 (PMC3582603; doi:10.1371/journal.pone.0056358)

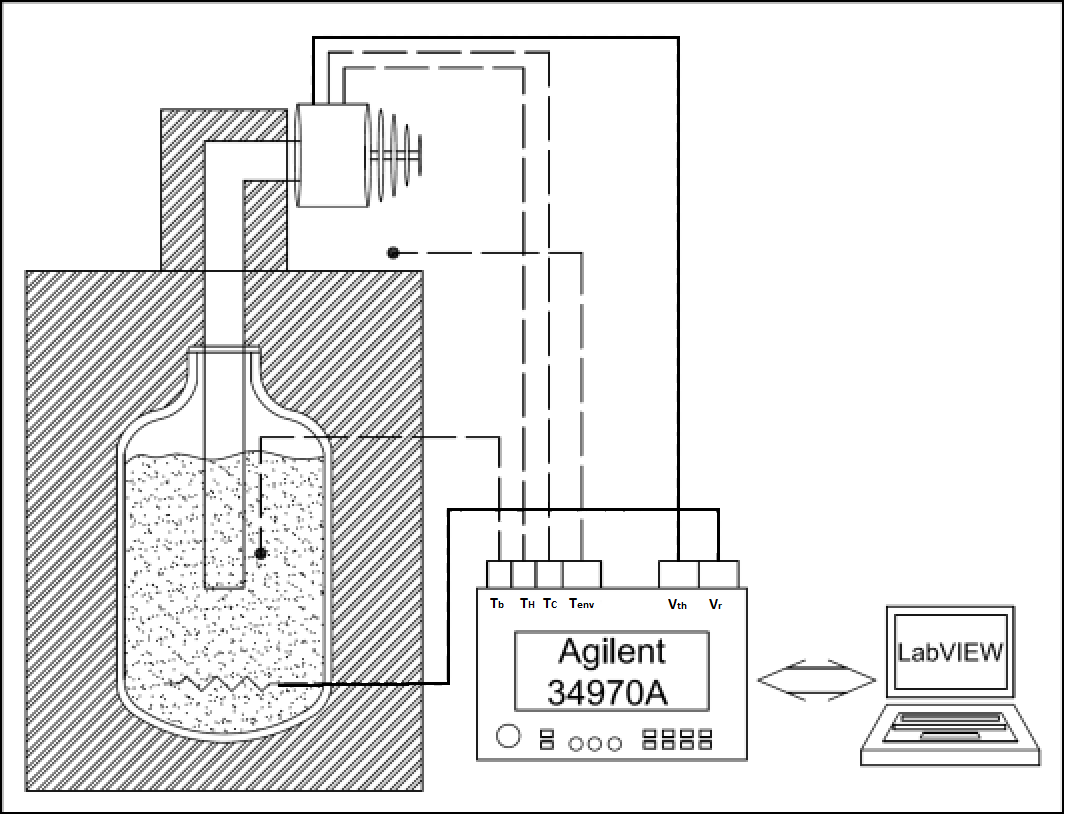

Supplement: Figure S1 — Schematic drawing of MTC data-recording system. Dashed lines represent thermocouple connections measuring the temperature of the broth (Tb), the temperature of the hot and cold sides of the thermogenerator (TH and TC, respectively), and the room temperature (Tenv); whereas continuous lines represent voltage measurements corresponding to the thermogenerator (Vth) and the electrical resistance (Vr). (TIF) [file pone.0056358.s001.tif]

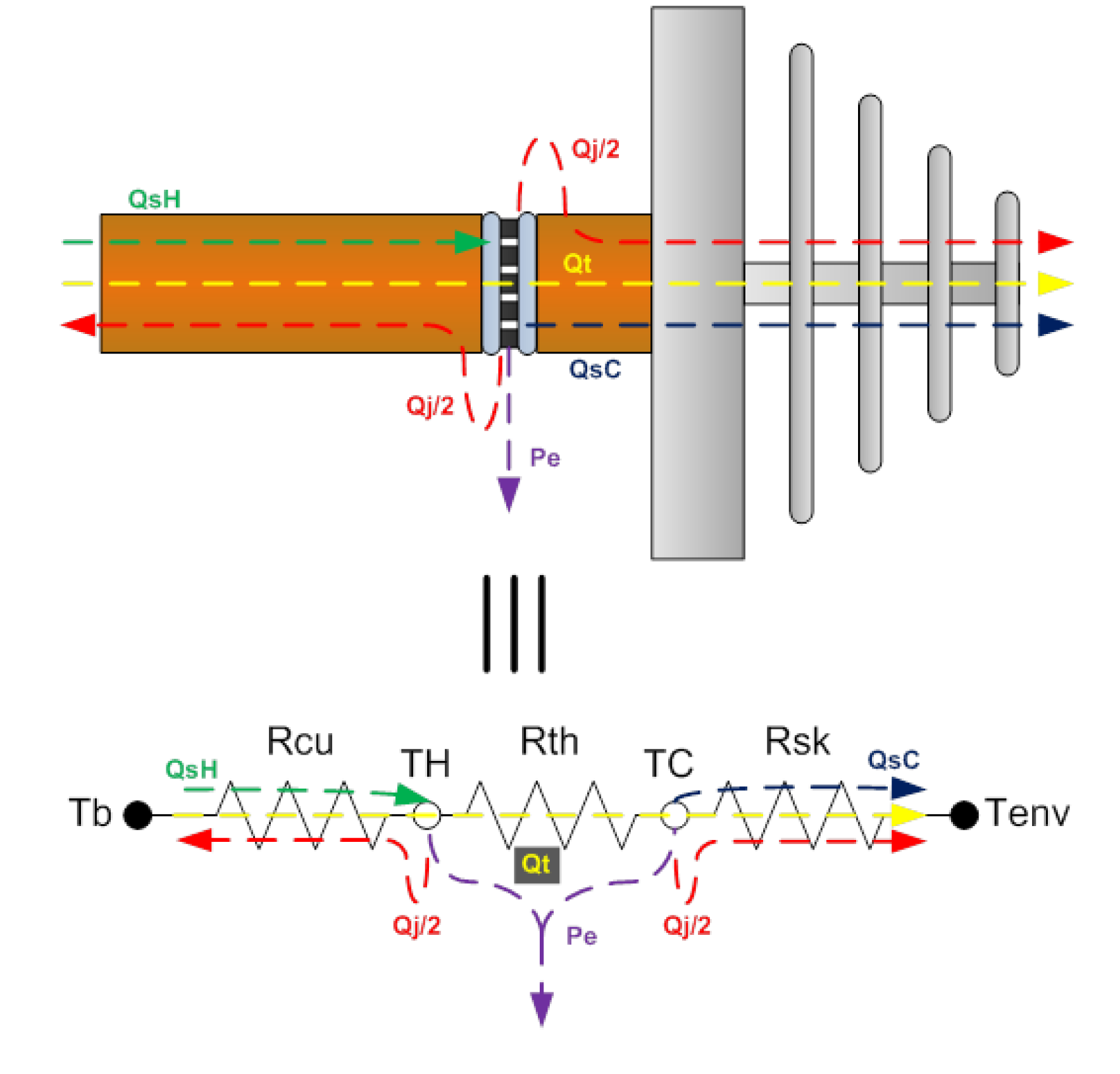

Supplement: Figure S2 — Schematic drawing of heat flows and resistances within the thermogenerator cell. Symbols used are in accordance with the nomenclature summarized in Table 1. (TIF) [file pone.0056358.s002.tif]
