# Supplementary material for: Towards a Microbial Thermoelectric Cell
Source: PLoS One. 2013 Feb 26;8(2):e56358. doi: 10.1371/journal.pone.0056358 (PMC3582603; doi:10.1371/journal.pone.0056358)
Supplement: Appendix S1 — Thermogenerator cell (MPG-D751) general equations. (DOCX) [file pone.0056358.s003.docx]

**Appendix S1. Thermogenerator cell (MPG-D751**) **general equations**

According to the first law of thermodynamics, the equations for a thermogenerator cell (for a schematic representation, see Fig. S2) are defined as follows [14]:

 (S1.1)

 (S1.2)

(S1.3)

Where *Q_H_* and *Q_C_* are the net heat flows (absorbed and released, respectively) through the hot and cold sides of the thermogenerator; *Q_sH_* and *Q_sC_* are the heat flows produced in the hot and cold sides of the cell due to the Seebeck effect (being *α* the Seebeck coefficient, and *T_H_* and *T_C_* the temperature of the hot and cold sides of the thermogenerator). *Q_j_* is the heat flow generated due to the Joule effect when the current (*I*) goes through the internal resistance (*R_i_*). This heat flow is equally distributed in both sides of the thermogenerator. *Q_t_* represents heat flow loss due to natural thermal conduction (being *R_th_* the thermal resistance) occurring between both sides of the cell, which are at a different temperature. *P_e_* represents the electrical power production on a load resistance (*R_load_*).

In order to obtain the maximum electrical power, the load resistance must be equal to the internal resistance of the thermogenerator. Thus, the electrical current of this circuit configuration is:

(S1.4)

Where *ΔT_th_* represents the difference in temperature between the hot and the cold side of the thermogenerator.

On the other hand, under an open-circuit configuration *I* is null, so there is no electrical power production, and both *Q_H_* and *Q_C_* are equal to *Q_t_*.
