# Supplementary material for: Towards a Microbial Thermoelectric Cell
Source: PLoS One. 2013 Feb 26;8(2):e56358. doi: 10.1371/journal.pone.0056358 (PMC3582603; doi:10.1371/journal.pone.0056358)
Supplement: Appendix S2 — TE-Power Probe model description. (DOCX) [file pone.0056358.s004.docx]

**Appendix S2. TE-Power Probe model description**

The definition of *I* for maximal power acquisition, Eq. S1.4 in Appendix S1, was taken into account to define a simple thermal model, in which every thermal resistance of the TE-Power Probe is considered (Fig. S2). The equations modeling TE-Power Probe performance are:

 (S2.1)

 (S2.2)

 (S2.3)

 (S2.4)

 (S2.5)

 (S2.6)

Where *V_o_* is the output voltage and *ΔT_th_* represents the difference in temperature between the hot and the cold side of the thermogenerator; *R_Cu_* is the thermal resistance of the cupper bar connecting the broth (at a temperature *T_b_*) and the hot side of the cell (at a temperature *T_H_*); and *R_Sk_* is the thermal resistance found between the cold side of the thermogenerator (*T_C_*) and the environment (considering room temperature *T_env_*).

Under an open-circuit configuration the model equations can be written as follows:

 (S2.7)

 (S2.8)

 (S2.9)

 (S2.10)

Where there is no electrical power production.
